# Echocardiographic Assessment of Left Ventricular Function in Three Oncologic Therapeutic Modalities in Women with Breast Cancer: The ONCO-ECHO Multicenter Study

**DOI:** 10.3390/jcm13092543

**Published:** 2024-04-26

**Authors:** Tomasz Gąsior, Beata Zaborska, Paweł Stachowiak, Małgorzata Sikora-Frąc, Katarzyna Mizia-Stec, Jarosław Kasprzak, Artur Bodys, Julia Bijoch, Adrianna Szmagała, Dariusz A. Kosior, Edyta Płońska-Gościniak

**Affiliations:** 1 Department of Cardiology and Structural Heart Diseases, Medical University of Silesia, 40-055 Katowice, Poland; 2 Boehringer Ingelheim International GmbH, 55218 Ingelheim, Germany; 3 Department of Cardiology, Centre of Postgraduate Medical Education, Grochowski Hospital, 04-073 Warsaw, Poland; 4 Department of Cardiology, Pomeranian Medical University, 70-111 Szczecin, Poland; 5 1st Department of Cardiology, School of Medicine in Katowice, Medical University of Silesia, 40-055 Katowice, Poland; kmizia-stec@sum.edu.pl; 6 Department of Cardiology, Medical University of Lodz, 90-419 Lodz, Poland; 7 Stefan Cardinal Wyszynski Regional Hospital, 20-468 Lublin, Poland; 8 Collegium Medicum—Faculty of Medicine, WSB University, 41-300 Dabrowa Gornicza, Poland; 9 Xth Department of Invasive Cardiology, Electrophysiology and Electrostimulation, American Heart of Poland, 43-100 Tychy, Poland; 10 Mossakowski Medical Research Centre Polish Academy of Sciences, 02-106 Warsaw, Poland

**Keywords:** cardio-oncology, breast cancer, chemotherapy, radiotherapy, echocardiography, cardiotoxicity, left ventricular diastolic dysfunction

## Abstract

**Background**: Oncological treatment of breast cancer may be associated with adverse effects on myocardial function. **Objectives**: The objective of this study was to compare the influence of three oncological treatment methods of intervention on the echocardiographic (ECHO) parameters of left ventricular function. **Materials and Methods**: One hundred and fifty-five women with breast cancer were divided into three groups depending on the type of therapy used: group I (AC)—anthracyclines; group II (AC + TZ)—anthracyclines + trastuzumab; and group III (RTls+)—anthracyclines with or without trastuzumab + left-sided radiotherapy. Prospective ECHO examinations were performed at baseline and every 3 months, up to 12 months from the start of the therapy. Patients with a history of chemotherapy or who were diagnosed with heart disease were not included in the study. **Results**: Out of 155 patients, 3 died due to cancer as the primary cause, and 12 withdrew their consent for further observation. Baseline systolic and diastolic ECHO parameters did not differ between the analyzed groups. Cardiotoxicity, according to the LVEF criteria, occurred during follow-up in 20 patients (14.3%), irrespective of the treatment method used. Diastolic echocardiographic parameters did not change significantly after 12 months in each group, except for the left atrial volume index (LAVi), which was significantly higher in the AC + TZ compared to the values in the RTls+ group. **Conclusions**: All three oncologic therapeutic modalities in women with breast cancer showed no significant differences in relation to the incidence of echocardiographic cardiotoxicity criterion; however, transient systolic decrease in LVEF was most frequently observed in the AC + TZ therapeutic regimen. Left-sided radiotherapy was not associated with excess left ventricular systolic and diastolic dysfunction during a 12-month follow-up period. The predictors of negative changes in diastolic parameters included age and combined anthracycline and trastuzumab therapy.

## 1. Introduction

Breast cancer is the most common malignancy in women in Poland and worldwide [1,2]. The contemporary treatment approach is complex, involving breast surgery, anthracycline-based chemotherapy (AC), and trastuzumab (TZ) for anti-human epidermal growth factor receptor 2 (HER-2)-positive patients. Radiotherapy (RT) is used when necessary, and hormone therapy is frequently utilized. Although the impact on the quality of life is meaningful, breast cancer has a relatively favorable prognosis.

Anthracyclines are potent broad-spectrum drugs used in many adjuvant and metastatic breast cancer treatments. The incorporation of TZ, a monoclonal antibody sequentially used following AC in cases of HER-2-positive breast cancer, was a major treatment advancement. However, there are data documenting an increased rate of cardiotoxicity in patients treated with AC and TZ [3,4].

Radiotherapy is a standard approach for localized breast cancer patients after breast-conserving surgery or in more advanced, metastatic diseases. Radiotherapy reduces local breast cancer relapses and improves survival but leads to a two-fold increase in late cardiovascular morbidity and mortality [5,6]. Radiation exposure induces microvascular injury, leading to myocardial inflammation, oxidative stress, and nonspecific diffuse progressive myocardial fibrosis [7,8]. The adverse effects of RT and chemotherapy are likely additive.

Echocardiography is a first-line diagnostic tool for cardiac monitoring during and after cancer therapy, particularly for the evaluation of left ventricular (LV) systolic function and the diagnosis of cardiotoxicity [9]. Diastolic dysfunction is an important predictor of all-cause mortality and plays an important role in the development of heart failure (HF). Some reports indicate an early onset of diastolic dysfunction even before the manifestation of contractile dysfunction in patients with breast cancer [10,11]. Conversely, other publications describing the effects of chemotherapy in breast cancer do not indicate any impact of the treatment on diastolic function [12], or the effect was only seen with high doses of anthracyclines [13]. Few reports have explored the impact of different types of breast cancer therapy on both the systolic and diastolic function of the left ventricle [11,13,14,15,16].

This study aimed to assess and compare the effects of three treatment approaches—anthracycline alone, anthracycline combined with trastuzumab, and/or left-sided radiotherapy—on echocardiographic parameters measuring the systolic and diastolic left ventricular function in breast cancer patients over a 12-month follow-up period.

We hypothesized that the effect of cardiac radiation in addition to contemporary chemotherapy may exert the most pronounced negative impact on LV ejection fraction and echocardiographic signs of LV filling in comparison to other treatment modalities.

## 2. Materials and Methods

### 2.1. Study Group and Design

This was an observational prospective cohort study carried out as part of the ONCO-ECHO multicenter registry. One hundred and fifty-five women (54.8 ± 9.8 years old) with histologically proven breast cancer from nine centers were prospectively enrolled in the study. The study protocol followed the ethical guidelines of the 1975 Declaration of Helsinki and was approved by the Ethics Committee of the Pomeranian Medical University in Szczecin. Written informed consent was obtained from all study participants.

Patients with a history of chemotherapy or RT prior to breast cancer diagnosis were not included in the study. Other exclusion criteria were age < 18 years old, baseline LV systolic dysfunction (ejection fraction [EF] < 55%), regional wall motion abnormalities at rest, LV hypertrophy (thickness of any segment > 13 mm), and significant valvular heart disease (defined as more than mild valvular regurgitation or stenosis).

Evaluations including medical history, physical examinations, resting electrocardiograms, routine laboratory tests, and transthoracic echocardiography (TTE) were performed at baseline (before therapy) and during follow-ups at three, six, nine, and twelve months.

Cancer therapy schemes, concomitant conditions, and cardiovascular medications were extracted from the medical records.

### 2.2. Therapy

According to clinical guidelines, patients underwent either breast conservation surgery or mastectomy and either sentinel node biopsy or axillary dissection.

In our multicenter study, the oncologic therapy was administered according to the local experience and clinical status of the patient. Patients were treated according to the AC, FAC, TAC, AT, FACT, EC, and ET regimens, and drug doses were individualized. All patients received AC chemotherapy, i.e., either doxorubicin (*n* = 142) or epirubicin (*n*-13), which was administered (4 or 6 cycles) as determined by the oncologist. The mean AC dose, expressed as the doxorubicin dose, was 255 ± 73.8 mg/m^2^. In addition, the patients received cyclophosphamide 89 (63%), docetaxel 79 (51%), and 5-fluorouracil 17 (11%). Fifty-four (34.8%) patients were treated with TZ. Endocrine therapy with tamoxifen was administered to 45 (29%) patients following completion of anticancer treatment.

Radiotherapy was indicated in patients after breast-conserving surgery and in those with advanced stages of the disease. During RT planning, a routine, pretreatment non-contrast computed tomography (CT) was performed. Attempts were made to minimize the dose to reduce the heat exposure during the RT treatment. RT was delivered using medial and lateral tangential photon beams of 6 MV and/or a mix of 6 MV and 18 MV. The treatment was performed with patients positioned supinely on an inclined board, with their arms extended above their heads. The radiotherapy regimen adhered to standard protocols, delivering either 50 Gy in 25 fractions or 42.4 Gy in 16 fractions utilizing a hypofractionated schedule. Patients undergoing breast-conserving surgery received an additional boost of 10–16 Gy delivered in 2 Gy fractions to the tumor bed.

The patients were divided into three groups based on the mode of therapy: the AC chemotherapy group; the AC + TZ group (AC chemotherapy followed by TZ); and the RTls+ group (AC chemotherapy with or without TZ and left-sided RT).

One hundred forty patients completed our observation: three patients died from primary cancer, and twelve patients withdrew their consent for further observation.

### 2.3. Echocardiogram

All patients underwent transthoracic echocardiography (TTE) with the use of a VIVID 9 ultrasound system (GE Healthcare) or Toshiba system. TTE was performed on initial admission prior to the chemotherapy initiation and was repeated 3, 6, and 12 months following enrollment. All echocardiograms were performed with the patient positioned in a left lateral position, and the images were obtained in the parasternal, apical, and subcostal views with simultaneous superimposed ECG. The measurements adhered to guidelines outlined by both the American Society of Echocardiography (ASE) and the Section of Echocardiography of the Polish Cardiac Society (SE PTK) [17,18]. Doppler blood flow measurements were obtained during passive end-expiration. Three consecutive cardiac cycles were sampled and averaged for all Doppler measurements. Myocardial velocities were assessed using tissue Doppler echocardiography, with a consistent radial sample volume dimension of 6 mm. Velocity was measured within a scale ranging from −20 to 20 cm/s, with three consecutive cardiac cycles averaged for each measurement. To evaluate left ventricular (LV) systolic function, the following assessments were conducted: measurement of end-diastolic diameter (LVEDd), end-systolic diameter (LVESd), interventricular septum dimension (IVSd), and posterior wall dimension (PWd) during end-diastole. Additionally, LV volumes were determined using the modified Simpson’s method, calculating end-diastolic volume (LVEDV) and end-systolic volume (LVESV), as well as ejection fraction. Systolic long-axis LV function was assessed by measuring peak systolic velocities of the septal and lateral mitral annulus (s’ septal and s’ lateral) using tissue Doppler echocardiography, along with mitral annular plane systolic excursion (MAPSE) derived from M-mode. To assess the LV diastolic function, we measured the parameters describing the early left ventricular filling phase and left atrium volume index. The peak early filling velocity (E) of mitral inflow was acquired by positioning the pulse-wave (PW) Doppler sample volume between the tips of the mitral leaflets in the apical 4-chamber view. The isovolumic relaxation time (IVRT) was assessed in the apical five-chamber view, where the PW Doppler sample volume was positioned between LV inflow and outflow, allowing simultaneous recording of the end of aortic ejection time and the onset of mitral inflow. Peak early diastolic velocity for the basal segments of the interventricular septum (e’ septal) and lateral wall (e’ lateral) was assessed in the apical 4-chamber view with tissue Doppler echocardiography. The E/e’ ratio was calculated by dividing E with the average of septal and lateral eʹ velocities. The left atrium (LA) diameter (LAd) was measured at end-systole in the parasternal long-axis view. The LA volume (LAV) was calculated in the apical 4-chamber and 2-chamber views using the biplane area-length method. The LAV index (LAVi) was defined as LA volume divided by the body surface area (BSA). The BSA was calculated using the Mosteller formula.

Due to technical challenges, particularly in patients post-left mastectomy, not all diastolic parameters were accessible for every patient. Regrettably, tricuspid regurgitation remained mild in the majority of cases, rendering it impractical to measure the maximum regurgitation velocity necessary for a comprehensive assessment of left ventricular diastolic dysfunction via echocardiography [19]. Nevertheless, the latest ESC guidelines on cardio-oncology only mention two parameters for evaluating left ventricular diastolic function: E/e’ and LAV (9). Cardiotoxicity was defined as a decrease in LVEF of >10 percentage points to a value < 50%, compared to the baseline value before the start of the oncological therapy.

### 2.4. Statistical Analysis

The results are expressed as the mean ± standard deviation or number and percentage. A *p*-value < 0.05 was considered statistically significant. Continuous variables were compared by paired or unpaired *t* tests. The chi^2^ test was used for the comparison of categorical variables. Two-way repeated-measures analysis of variance (ANOVA) was performed to investigate differences in the assessed variables within patients across the five time points (baseline and at the 3-, 6-, 9-, and 12-month follow-ups). The prognosis of the E/e’ ratio and LAVi changes over time (between baseline and the 12-month follow-up) were tested with a multivariate linear regression analysis model. The multivariate model was developed with stepwise inclusion and exclusion at a significance level of 0.15. Age, history of diabetes, arterial hypertension, body mass index, AC dose, and therapy regimen (AC or AC + TZ or RTls+) were assessed. The analyses were carried out using SAS 9.2 software.

## 3. Results

### 3.1. Clinical Characteristics

One hundred fifty-five patients were included in the study. Baseline, clinical characteristics regarding vascular risk factors, comorbidities, medications, and the oncological characteristics of the enrolled patients are summarized in Table 1. The patients were divided into three groups based on the mode of the received anticancer therapy: the AC group—63 patients; the AC + TZ group—27 patients; and the RTls+ group—65 patients. In the group treated with left-sided RT, 38 (58%) patients received AC alone, and 27 (42%) patients received AC with subsequent TZ. Right-sided RT was added to the therapy regimen in 28 (44%) patients from the AC group and in 11 (41%) patients from the AC + TZ group.

The baseline variables were similar among the groups of different therapy regimens.

The anthracycline dose was slightly higher in the RTls+ group than in the other groups.

All patients showed normal LV volumes, well-preserved LV systolic function described as LVEF, MAPSE, myocardial systolic velocities, indices of early left ventricular filling, and left atrial volume (Table 2). Only MAPSE was significantly higher in the RTls+ group compared to the AC group.

### 3.2. Echocardiographic Results

During the follow-up period, cardiotoxicity, as defined by the ESC criteria [9] (based on the LVEF changes), occurred in 20 patients (14.3%) and was evident in 7 patients after 3 months (3 AC; 2 AC + TZ; 2 RTl+), in 7 patients after 6 months (2 AC; 3 AC + TZ; 1 RTl+), in 5 patients after 9 months (1 AC; 2 AC + TZ; 2 RTl+), and in 2 patients after 12 months of therapy (1 AC; 1 RTl+). Although no statistically significant disparity in the occurrence of cardiotoxicity was detected across various treatment modalities, its incidence was notably higher in patients undergoing AC + TZ therapy, observed in 22% of cases. The average LVEF values for the entire study group during follow-up are shown in Table 3. In patients meeting the criteria for cardiotoxicity, 19 had a decrease in LVEF to 44–49% only during one of the examination visits, and in 1 patient, LV systolic dysfunction was present both at the 3 and 6 months which returned to normal value in the examination after 12 months.

The changes in the diastolic parameter values across three treatment modalities were examined at baseline and at 3, 6, 9, and 12 months during the follow-up period, as shown in Table 4. While comparisons revealed minimal discrepancies in the analyzed parameters’ values, statistically significant differences were observed during certain intervals of the observation period. Nevertheless, after 12 months, only the left atrial volume index (LAVi) demonstrated a significant increase in the AC + TZ group compared to the RTIs+ group. 

Analysis of variance (ANOVA) with repeated measurements for IVRT changes in the whole group revealed significant differences regarding time (*p* = 0.009) and type of treatment (*p* = 0.004). A shorter IVRT was observed after 3 and 9 months and in patients treated with AC + TZ. However, after 12 months, IVRT values did not differ with respect to baseline and method of treatment. No significant differences were found in e’ septal, e’ lateral, or E/e’ for both time (*p* = 0.85, *p* = 0.80, and *p* = 0.55, respectively) and type of treatment (*p* = 0.22, *p* = 0.37, and *p* = 0.80, respectively). No significant differences in relation to the incidence of the abovementioned variables were found among different treatment regimens. During the initial examination, an abnormal E/e’ value (>14), indicative of elevated filling pressure, was documented in four patients. After a 12-month follow-up, this anomaly persisted in five patients, distributed as follows: two in the AC group, two in the AC + TZ group, and one in the RTIs+ group.

The most pronounced relative differences among patients receiving different modes of therapy were found in relation to LAVi (*p* = 0.031) (Table 4). Notably, at baseline, LAVi did not differ among the groups. The AC + TZ group showed LA dilation compared to the other groups at the 3-, 6-, and 12-month follow-ups. LAVi increased significantly between baseline and the 12-month follow-up in patients receiving AC alone or AC + TZ (*p* = 0.028 and *p* = 0.048, respectively). This difference was not observed in the RTls+ patients. At 12 months, LAVi > 34 mL/m^2^ was newly observed in one patient from the AC group and in three patients from the AC + TZ group. No such case was observed in the RTls+ group.

According to multivariate stepwise regression analysis, age was shown to be an independent predictor of changes in the E/e’ ratio between baseline and the 12-month follow-up (Table 5).

On the other hand, anthracycline dose and combined AC + TZ therapy were shown to be independent predictors of changes in the LAVi between baseline and the 12-month follow-up (Table 5).

The overall prevalence of diastolic parameters deterioration was moderate, and no significant differences were found among groups with different modes of therapy.

## 4. Discussion

The contemporary protocols of chemotherapy and radiotherapy in breast cancer treatment are linked to potential adverse effects on cardiac and arterial functions. Accordingly, the existing guidelines from the European Society of Cardiology (ESC) advocate for regular cardiac evaluations for patients undergoing these treatments [9]. While criteria for diagnosing cardiotoxic effects on systolic heart function have been established, recommendations and cutoff criteria for diagnosing iatrogenic diastolic dysfunction remain undefined. Recent studies suggest the superiority of global longitudinal strain (GLS) assessment over left ventricular ejection fraction (LVEF) assessment in diagnosing ‘toxic’ systolic dysfunction, prognosticating subclinical cardiotoxicity, and monitoring heart failure during anthracycline treatment [11,14,15,20,21]. Diastolic dysfunction is one of many potential complications of cancer therapy and may cause major cardiac morbidity such as dyspnea on mild exertion, fatigue, and fluid retention. Conventional assessment of LV diastolic function should be added to the assessment of LV systolic function during and after the therapy [22]. Among the examined parameters, global longitudinal strain (GLS), E/e’, and LAVi have been identified as valuable tools for assessing left ventricular diastolic function. Distinguishing between normal and abnormal diastolic function through noninvasive measurements poses a significant challenge. Currently, in patients with preserved LV ejection function (LVEF > 50%), four recommended variables are useful as follows: annular e’ velocity, average E/e’ ratio, LA maximum volume index (LAVi), and peak tricuspid valve regurgitation velocity (pTRv). The cut-offs for the abnormal values are as follows: e’ (septal < 7 cm/s; lateral < 10 cm/s), E/e’ > 14, LAVi > 34 mL/m^2^, and pTRv velocity > 2.8 m/s. Diastolic dysfunction is present if more than half of the available parameters meet these cut-off values [19], and this algorithm may be useful in assessing the diastolic dysfunction grade. However, in the majority of original papers and meta-analyses concerning the assessment of left ventricular diastolic function during and after chemo- and radiotherapy, authors often refrain from presenting a comprehensive diastolic assessment for grading diastolic dysfunction. Instead, they typically focus on utilizing the most practical and clinically relevant parameters that are easy to register and measure [12,13,14,23,24].

The study aimed to compare the effects of three treatment modalities on left ventricular (LV) function and to investigate the hypothesis that adding cardiac radiation to contemporary chemotherapy in breast cancer patients may exert a more detrimental impact on LV ejection fraction and indices of LV filling compared to other treatment methods. From a pathophysiological point of view, it has been suggested that RT induces inflammation and tissue fibrosis and therefore can lead to increased LV wall stiffness and can ultimately cause impaired LV relaxation and filling [25]. In the existing literature, some conflicting reports have been published on the effects of RT on diastolic function, while the incidence rates depend on the investigational methods used and the time delay.

Sritharan et al. [26], in a 6-week observation of 40 patients following radiotherapy for breast cancer treatment, reported no changes in LV diastolic function parameters. In contrast, Skytta et al. demonstrated, in a 3-year follow-up post-adjuvant radiotherapy, not only deterioration in left ventricular ejection fraction (LVEF) and global longitudinal strain (LVGLS), but also prolongation of isovolumic relaxation time (IVRT), reduction in E-wave velocity, and an increase in left atrial volume index (LAVi). These changes were more pronounced in cases of left-sided cancer location [24].

In our study, we described only minor changes and identified a few new cases of diastolic dysfunction during the 12-month follow-up period. No increase in the incidence of LV diastolic dysfunction due to added left-sided RT was observed. Only a few cases of elevated filling pressure indicated by E/e’ > 14 were found in the study group. According to multivariate stepwise regression analysis, left-sided RT was not shown to be an independent predictor for changes in either the E/e’ ratio or the LAVi. Age was the independent predictor of changes in the E/e’ ratio between baseline and the end of the 12-month observation. We acknowledge that the patients were observed over a 12-month follow-up period, which may be considered relatively short for capturing changes related to fibrosis. In a population-based, case–control study of older women with 59 patients and 111 controls who underwent contemporary RT for breast cancer between 1998 and 2013, the mean interval from RT to HF was 5.8 ± 3.4 years. The odds ratio (95% CI) for any HF per log mean cardiac radiation dose was 9.1 (3.4–24.3); it was 16.9 (3.9–73.7) for HF with preserved EF and 3.17 (0.8–13.0) for HF with reduced EF [27].

Marinko et al. observed 84 patients in a left-breast cancer group and 91 in a right-breast cancer group, who underwent chemotherapy, with some patients also receiving radiotherapy (45% and 32%, respectively), over a median follow-up period of 57 months (range: 37–71). Among the patients included in the study, 42% exhibited left ventricular diastolic dysfunction upon follow-up, predominantly classified as mild. None of the patients had severe diastolic dysfunction described. However, it is important to note that the baseline diastolic function was not assessed. The authors concluded that RT had no significant influence on LV function [28]. Skytta T et al. reported prolongation of deceleration time in patients in an early disease stage and in left-sided breast cancer patients receiving adjuvant breast RT without prior chemotherapy, especially in the group with an increase in high-sensitivity cardiac troponin T. Other standard echocardiographic and tissue Doppler parameters of diastolic function remained unchanged [24].

The study performed by Cao L et al. revealed a high prevalence of LV diastolic dysfunction in both patients treated with concurrent TZ and RT and left-sided RT alone at the 6-month follow-up [29]. The diagnosis of diastolic dysfunction was based on an E/e’ ratio ≥ 15, and in the group with an E/e’ ratio of 8–15, accessory criteria based on the evolution of the mitral inflow profile were used.

Left atrial volume index (LAVi) offers valuable insights into left ventricular diastolic function and the chronicity of the disease [19]. We observed a significantly higher left atrial volume index (LAVi) in patients treated with AC chemotherapy followed by TZ compared to those receiving other modes of therapy throughout the entire follow-up period. We also found that LAVi increased significantly between baseline and the 12-month follow-up in patients receiving AC chemotherapy alone or AC chemotherapy with TZ. This result is in concordance with the existing data from the literature [30,31].

In a study by Calle MCA et. al., the combined AC + TZ therapy resulted in a high rate of cardiotoxicity reaching 20% of patients [20]. In a longitudinal cohort study by Upshaw et al. involving 362 breast cancer participants, abnormal diastolic function was documented in 80% of patients treated with doxorubicin or doxorubicin followed by trastuzumab during a 3-year follow-up [10]. However, these changes were not observed in patients treated with trastuzumab alone. The authors suggest subsequent deterioration in systolic function among patients with previous post-chemotherapy diastolic dysfunction. Over a 6.5-year follow-up period, they observed cancer therapy-related cardiac dysfunction (CTRCD), characterized by a decline in left ventricular ejection fraction (LVEF) of >10% to <50%, in 17% of the entire cohort. However, this occurrence was notably higher, at 32%, in the doxorubicin plus trastuzumab group. In our study, during a 1-year follow-up, CTRCD was observed in 13% of all patients, with the highest percentage noted in the AC + TZ group (22%). However, due to the relatively small number of study participants in each subgroup, no statistically significant differences were found between the subgroups.

The utilization of intrinsic wave velocity propagation, a well-established parameter for assessing the dynamics of inflow into the left ventricle, proves to be a valuable tool for the early detection of subclinical LV diastolic dysfunction in breast cancer patients [32].

In our study, multivariate stepwise regression analysis revealed a relationship between anthracycline dose and TZ therapy with changes in the left atrial volume index (LAVi) between baseline and the 12-month follow-up. However, it is important to note that changes in LAVi during treatment may also be indicative of fluid retention. 

## 5. Limitations of the Study

The protocol for this study was designed based on the hypothesis that cardiac radiation, in addition to contemporary chemotherapy, might increase the incidence of left ventricular (LV) diastolic dysfunction. The study group comprised patients treated with either AC alone or AC followed by TZ, with potential differences anticipated between these groups. Longer follow-up periods and further investigations may be necessary to validate these results. A notable limitation in assessing systolic function during oncological therapy is the inability to evaluate the longitudinal deformation of the left ventricle, which was not feasible during patient consultations in oncology wards. However, it is important to note that this parameter is not mandatory for cardiotoxicity assessment.

To some extent, the interpretation of the obtained results is limited by the lack of assessment of possible changes in the intensity of the oncological chemotherapy protocol. A complete assessment of diastolic function, according to currently accepted recommendations, was not feasible in some patients due to challenges with adequate visualization. This limitation highlights the difficulties encountered in implementing multiparameter assessments in real-world circumstances.

## 6. Conclusions

All three oncologic therapeutic modalities in women with breast cancer did not differ significantly with respect to the incidence of echocardiographic cardiotoxicity criterion; however, transient systolic decrease in LVEF was most frequently observed in the AC + TZ therapeutic regimen.

The prevalence of deterioration in diastolic parameters is moderate regardless of the mode of therapy. The predictors of negative changes in diastolic parameters included age and combined anthracycline and trastuzumab therapy. Left-sided radiotherapy was not associated with increased LV systolic and diastolic dysfunction during the 12-month follow-up period.

## Figures and Tables

**Table 1:** Baseline characteristics of the total population and subgroups.

Characteristic	Total *n* = 155	AC *n* = 63	AC + TZ *n* = 27	RTls+ *n* = 65	*p*-Value
Age, mean (SD)	54.8 ± 9.8	55.1 ± 10.3	56.1 ± 9.7	53.9 ± 9.4	0.60
Weight, mean (SD), kg	69.3 ± 11.1	70.4 ± 11.7	68.6 ± 11.2	68.4 ± 9.4	0.58
Body mass index, mean (SD), kg/m^2^	26.4 ± 4.1	26.7 ± 4.2	26 ± 3.9	26.4 ± 4	0.79
Tumor side Left side, N (%) Right side, N (%)	85 (54) 71 (45)	15 (24) 48 (76)	5 (19) 22(81)	65 (100) 1 (1)	
Ca ductale	67 (43)	29 (46)	13 (48)	25 (38)	0.92
Therapy regimen					
Anthracycline dose, mean (SD), mg/m^2^	255 ± 73.8	255 ± 66.8	225.6 ± 56.2	266.7 ± 84.4	0.06
Anthracycline dose > 240 mg/m^2^, N (%)	51 (33)	21 (33)	5 (19)	25 (38)	0.21
Trastuzumab, N (%)	54 (35)	0	27 (100)	27 (42)	<0.0001
Docetaxel, N (%)	79 (51)	35 (56)	12 (44)	32 (49)	0.59
5-FU, N (%)	17 (11)	5 (8)	1 (4)	11 (17)	0.11
Tamoxifen, N (%)	45 (29)	17 (27)	11 (41)	17 (26)	0.08
Radiotherapy, N (%) Left side, N (%) Right side, N (%)	65(42) 40(26)	0 28 (44)	0 11 (41)	65 (100) 1 (1)	<0.0001
Vascular risk factors					
Smoking	46 (30)	21 (33)	5 (19)	20 (31)	0.35
Arterial hypertension	67 (43)	28 (44)	11 (41)	28 (44)	0.89
Diabetes	9 (6)	2 (3)	3 (11)	4 (6)	0.28
Stroke	2 (1)	2 (3)	0	0	0.67
Medication					
Beta-blockers	45 (29)	20 (32)	5 (19)	20 (31)	0.36
ACEI/ARB	35 (23)	17 (27)	8 (30)	10 (15)	0.29
Statins	24 (15)	11 (17)	5 (19)	8 (12)	0.84

AC, anthracyclines; AC + TZ, anthracyclines + trastuzumab; RTls+, left-sided radiotherapy andanthracyclines with or without trastuzumab; 1 patient with left and right breast cancer.

**Table 2:** Baseline left ventricular echocardiographic parameters.

	Total	AC	AC + TZ	RTls+	*p*-Value
LVEF (%)	63.0 ± 5.2	63.0 ± 5.4	63.0 ± 5.4	63.0 ± 5.0	0.97
LVEDd (mm)	44.9 ± 5.2	44.6 ± 4.9	45.8 ± 5.5	44.7 ± 5.3	0.58
LVESd (mm)	27.6 ± 5.1	26.7 ± 4.4	27.8 ± 5.6	28.3 ± 5.6	0.20
LVEDV (mL/m^2^)	75.6 ± 20.2	78.1 ± 23.1	73.2 ± 20.1	73.2 ± 15.2	0.49
LVESV (mL/m^2^)	27.4 ± 8.7	28.6 ± 10.6	25.8 ± 7.2	26.4 ± 6.1	0.38
s’ septal (cm/s)	7.5 ± 1.8	7.8 ± 2.0	7.7 ± 1.6	7.2 ± 1.6	0.13
s’ lateral (cm/s)	8.0 ± 2.5	8.3 ± 2.4	8.1 ± 3.2	7.7 ± 2.4	0.45
MAPSE (mm)	16.3 ± 3.0	15.9 ± 3.0	17.4 ± 2.9	17.6 ± 2.8	0.023 *
e’ septal (cm/s)	9.1 ± 2.7	8.8 ± 2.6	8.5 ± 2.0	9.6 ± 2.9	0.15
e’ lateral (cm/s)	10.8 ± 3.6	11.0 ± 3.6	11.5 ± 3.0	10.4 ± 3.9	0.50
E/e’	8.0 ± 2.9	7.8 ± 2.5	7.8 ± 2.2	8.3 ± 3.4	0.70
IVRT (ms)	95 ± 20	94 ± 20	95 ± 20	97 ± 20	0.2
LAVi (mL/m^2^)	26 ± 8	24 ± 9	29 ± 8	27 ± 6	0.16

AC, anthracyclines; AC + TZ, anthracyclines + trastuzumab; RTls+, left-sided radiotherapy and anthracyclines with or without trastuzumab; LVEF, left ventricular ejection fraction; LVEDd, left ventricular end-diastolic diameter; LVESd, left ventricular end-systolic diameter; LVEDV, left ventricular end-diastolic volume; LVESV, left ventricular end-systolic volume; MAPSE, mitral annulus plane systolic excursion; s’, septal-peak systolic septal velocity; s’, lateral-peak systolic lateral velocity; IVRT, isovolumic relaxation time; LAVi, left atrial volume index; * AC vs. AC + TZ: 0.16, AC vs. RTls+: 0.018, and AC + TZ vs. RTls+: 0.16.

**Table 3:** Left ventricular ejection fraction at baseline and during follow-up in all patients.

	Baseline	3 Months	6 Months	9 Months	12 Months	*p*-Value
LVEF (%) + SD	63.0 ± 5.2	61.3 ± 6.6	61.0 ± 7.0	59.5 ± 4.7	62.6 ± 10.9	NS

NS—non-significant.

**Table 4:** Serial echocardiographic parameters of diastolic function at baseline and during follow-up.

Variable	Total n = 155	AC n = 63	AC + TZ n = 27	RTls+ n = 65	*p*-Value
IVRT (ms):					
baseline 3 months 6 months 9 months 12 months	95 ± 20 92 ± 18 95 ± 20 92 ± 17 95 ± 19	94 ± 20 91 ± 20 97 ± 18 88 ± 16 92 ± 15	95 ± 20 89 ± 23 87 ± 25 87 ± 19 90 ± 15	97 ± 20 94 ± 15 97 ± 18 98 ± 16 100 ± 23	0.2 0.036 * 0.101 **0.002 #** 0.16
e’ septal (cm/s):					
baseline 3 months 6 months 9 months 12 months	9.1 ± 2.7 8.7 ± 2.4 8.8 ± 2.3 8.7 ± 2.3 8.9 ± 2.5	8.8 ± 2.6 8.0 ± 1.9 8.8 ± 2.5 8.4 ± 2.1 8.5 ± 2.5	8.5 ± 2.0 8.9 ± 2.3 9.4 ± 2.3 9.5 ± 2.2 9.5 ± 2.6	9.6 ± 2.9 9.2 ± 2.7 8.5 ± 2.1 8.8 ± 2.4 9.1 ± 2.6	0.15 0.032 ** 0.31 0.18 0.23
e’ lateral (cm/s):					
baseline 3 months 6 months 9 months 12 months	10.8 ± 3.6 10.5 ± 3.1 10.5 ± 3.4 10.7 ± 3.3 10.5 ± 3.1	11.0 ± 3.6 10.2 ± 2.9 10.2 ± 3.4 10.6 ± 2.9 9.9 ± 2.7	11.5 ± 3.0 11.2 ± 3.5 11.6 ± 3.8 11.1 ± 3.5 11.2 ± 3.4	10.4 ± 3.9 10.4 ± 3.3 10.4 ± 3.2 10.6 ± 3.5 10.7 ± 3.3	0.50 0.49 0.29 0.77 0.18
E/e’:					
baseline 3 months 6 months 9 months 12 months	8.0 ± 2.9 8.1 ± 2.6 7.9 ± 2.5 8.2 ± 2.4 8.2 ± 2.7	7.8 ± 2.5 8.3 ± 2.5 8.4 ± 2.7 8.1 ± 2.4 8.4 ± 2.8	7.8 ± 2.2 8.1 ± 3.3 7.5 ± 2.5 8.3 ± 2.8 8.2 ± 3.0	8.3 ± 3.4 9.0 ± 2.4 7.6 ± 2.2 8.3 ± 2.3 7.9 ± 2.6	0.70 0.79 0.17 0.91 0.65
LAVi (mL/m^2^):					
baseline 3 months 6 months 9 months 12 months	26 ± 8 25 ± 7 27 ± 8 27 ± 9 28 ± 8	24 ± 9 25 ± 6 26 ± 7 26 ± 11 27 ± 8	29 ± 8 31 ± 8 33 ± 12 30 ± 8 34 ± 9	27 ± 6 24 ± 6 25 ± 6 26 ± 6 26 ± 7	0.16 **0.011 ##** 0.029 *** 0.51 **0.040 ###**

AC, anthracyclines; AC + TZ, anthracyclines + trastuzumab; RTls+, left-sided radiotherapy and anthracyclines with or without trastuzumab; IVRT, isovolumic relaxation time; s’, septal-peak systolic septal velocity; s’, lateral-peak systolic lateral velocity; LAVi, left atrial volume index. The comparisons among all groups (ANOVA) are reflected by the *p*-values provided in the table. Significant univariate differences are marked in the table and are provided below: * AC vs. AC + TZ: 0.045, AC + TZ vs. RTls+: 0.039; # AC vs. AC + TZ: 0.021, and AC + TZ vs. RTls+: 0.001; ** AC vs. RTls+: 0.028; ## AC + TZ vs. RTls+: 0.018 and AC + TZ vs. RTls+: 0.013; *** AC vs. AC + TZ: 0.038 and AC + TZ vs. RTls+: 0.036; ### AC + TZ vs. RTls+: 0.037.

**Table 5:** Multivariate stepwise regression analysis for changes in the E/e’ ratio and LAVi between baseline and the 12-month follow-up.

Variable	Multivariate Analysis		
	Mean Estimator ± SE	Partial R^2^	*p*-Value
Change in E/e’ ratio			
Age	0.06 ± 0.02	0.06	0.014
Arterial hypertension	0.69 ± 0.47	0.01	0.147
Change in LAVi			
Anthracycline dose	0.03 ± 0.01	0.03	0.031
TZ vs. AC	3.87 ± 1.76	0.03	0.030
Diabetes	−5.51 ± 3.17	0.02	0.086
Age	0.11 ± 0.06	0.02	0.089

LAVi, left atrial volume index; TZ, trastuzumab; AC, anthracycline.

## Data Availability

The datasets used and/or analyzed during the current study are available from the corresponding author upon reasonable request.

## List of Abbreviations

AC	anthracycline chemotherapy
AC regimen	doxorubicin + cyclophosphamide
ANOVA	analysis of variance
ASE	American Society of Echocardiography
AT regimen	doxorubicin + paclitaxel or docetaxel
BSA	body surface area
CT	computed tomography
E	peak of early filling velocity of mitral inflow
EC regimen	epirubicin + cyclophosphamide
EF	ejection fraction
e’ septal	peak early diastolic septal velocity
e’ lateral	peak early diastolic lateral velocity
ET regimen	epirubicin + paclitaxel or docetaxel
FAC regimen	fluorouracil + doxorubicin + cyclophosphamide
FACT regimen	fluorouracil + doxorubicin + cyclophosphamide + paclitaxel or docetaxel
HER-2	anti-human epidermal growth factor receptor 2
HF	heart failure
IVRT	isovolumic relaxation time
IVSd	interventricular septum dimension
LA	left atrium
LAd	left atrium diameter
LAV	left atrium volume
LAVi	left atrium volume index
LV	left ventricular
LVEDd	left ventricular end-diastolic diameter
LVEDV	left ventricular end-diastolic volume
LVESd	left ventricular end-systolic diameter
LVESV	left ventricular end-systolic volume
LVDD	left ventricular diastolic dysfunction
MAPSE	mitral annulus plane systolic excursion
PWd	posterior wall dimension
RT	radiotherapy
RTls+	anthracycline chemotherapy with or without trastuzumab and left-sided radiotherapy
SE PTK	Section of Echocardiography of Polish Cardiac Society
s’ septal	peak systolic septal velocity
s’ lateral	peak systolic lateral velocity
TAC regimen	docetaxel + doxorubicin + cyclophosphamide
TTE	transthoracic echocardiography
TZ	trastuzumab
